# History of a Case of Carcinoma of the Antrum*From the Radium and Oncologic Institute of Los Angeles.

**Published:** 1923-03

**Authors:** George W. Brown

**Affiliations:** Los Angeles


### HISTORY OF A CASE OF CARCINOMA OF THE ANTRUM *

BY DR. GEORGE W. BROWN, LOS ANGELES

Patient Mr. J. A. received a blow in the nose with a hammer, in 1919, from which time he dates his trouble. In June, 1921, he had neuralgia on the side of his face and had a couple of teeth extracted, but did not get any better. Later he had a radiogram taken, though no pathologic condition was found. Then experienced a burning sensation in the lip, nose and cheek and a thin watery discharge came from the nose.

On November 29, 1921, he called at my office and on examination I found the right side of his nose occluded, caused by a deviation of the nasal septum, and his right eye was almost swollen shut and he was suffering great pain in the region of the maxillary sinus. On December 20, 1921, I did a sub-mucous resection of the nasal septum at the California Lutheran Hospital. The right eye, although much better, was still swollen.

On December 27, 1921, I did a radical antrum operation, Caldwell-Luc, at the California Lutheran Hospital, and removed an amount of what appeared to be cancerous tissue and resected the infra-orbital nerve, which relieved his pain considerably. The laboratory findings of the specimen was carcinoma of the antrum. I then referred the patient for radium treatment and on February 2, 1922, I made an opening in the roof of his mouth, on the right side, the opening leading directly into the antrum. Radium treatments were taken from February 3 to April 13, 1922. By May 26, 1922, all evidence of the disease had disappeared and the patient is today greatly improved in health.

The report on the above case from the Radium and Oncologic Institute of Los Angeles was as follows: An

* From the Radium and Oncologic Institute of Los Angeles.

opening was made into the antrum through the roof of the mouth about 2 cm. in diameter, opposite the second molar. The antrum was found to be filled with carcinomatous tissue. Through this opening radium was introduced into the cavity and 2,153 Millicurie hours were given equally disposed throughout the cavity of the antrum between February 3 and April 13, 1922. On May 26, 1922, all evidence of the disease had disappeared, leaving the antral cavity communicating with the nasal cavity and with the mouth through the surgical opening. Patient was recommended to visit a dentist for prosthetic appliance.

